# When Tuberculosis Left the Heart in a Cage: Multimodality Imaging Revealing Constrictive Pericarditis in a Young Adult

**DOI:** 10.7759/cureus.115722

**Published:** 2026-09-03

**Authors:** Nadia Loudiyi, Hafsa Erregui, Mohamed Malki, Najat Mouine, Zouhair Lakhal

**Affiliations:** 1 Cardiology, Mohammed V University, Rabat, MAR; 2 Cardiac Catheterization, Mohammed V University, Rabat, MAR

**Keywords:** cardiac magnetic resonance, constrictive pericarditis, effusive-constrictive physiology, multimodality imaging, mycobacterium tuberculosis, tuberculous pericarditis

## Abstract

Tuberculous pericarditis (TBP) is a potentially severe manifestation of extrapulmonary tuberculosis that may lead to pericardial effusion and constrictive physiology and, in some patients, progress to fixed constrictive pericarditis. Diagnosis can be particularly challenging in young immunocompetent patients without overt pulmonary involvement. We report the case of a 20-year-old immunocompetent man presenting with a one-month history of weight loss, profuse night sweats, nocturnal fever, and typical pericarditic chest pain. Clinical examination revealed fever and signs of systemic venous congestion. Transthoracic echocardiography demonstrated marked pericardial thickening, moderate circumferential effusion, septal bounce, approximately 25% respiratory variation in transmitral flow, annulus reversus, and annulus paradoxus, supporting the presence of pericardial effusion with constrictive physiology. Contrast-enhanced computed tomography confirmed diffuse pericardial thickening and effusion without calcification or pulmonary parenchymal involvement. At the same time, cardiac magnetic resonance demonstrated intense late gadolinium enhancement of the thickened pericardium, supporting active pericardial inflammation. Xpert Mycobacterium tuberculosis/Rifampicin assay (Xpert MTB/RIF; Cepheid, Sunnyvale, CA, USA) testing of the pericardial fluid detected Mycobacterium tuberculosis, subsequently confirmed by positive mycobacterial culture. Following multidisciplinary discussion, a six-month antituberculous regimen combined with adjunctive corticosteroid therapy was initiated, with close clinical and multimodality imaging follow-up and reassessment for pericardiectomy according to clinical evolution. This case highlights the complementary value of echocardiography, computed tomography, and cardiac magnetic resonance in characterizing tuberculous pericardial disease associated with constrictive physiology and defining its inflammatory substrate. Identification of active pericardial inflammation in the presence of constrictive physiology may indicate a potentially reversible component; however, actual reversibility requires confirmation during longer-term follow-up.

## Introduction

Tuberculous pericarditis (TBP) represents a clinically significant and potentially life-threatening manifestation of extrapulmonary tuberculosis, accounting for approximately 1-2% of extrapulmonary tuberculosis cases, with a substantially greater burden in tuberculosis-endemic regions [1,2]. Persistent pericardial inflammation may lead to pericardial effusion and constrictive physiology and, in some patients, progress to fixed constrictive pericarditis, making early recognition and appropriate treatment clinically important [1].

The diagnosis becomes particularly challenging in young immunocompetent patients without overt pulmonary tuberculosis, in whom constitutional symptoms and pericardial manifestations may lack specificity [2]. In this setting, multimodality cardiovascular imaging is pivotal. Echocardiography reveals the physiological features suggestive of constriction, computed tomography (CT) accurately defines pericardial morphology, while cardiac magnetic resonance (CMR) provides unique tissue characterization by identifying active pericardial inflammation [1,3]. Beyond diagnosis, this distinction is clinically relevant, as recognition of an active inflammatory phenotype associated with constrictive physiology may identify a potentially reversible component and inform therapeutic decision-making [1,3].

We report the case of a 20-year-old immunocompetent man with microbiologically confirmed TBP, no known tuberculosis exposure, and no radiological evidence of pulmonary involvement, presenting with pericardial effusion and echocardiographic evidence of constrictive physiology. The particular interest of this case lies in the comprehensive multimodality demonstration of both active pericardial inflammation and constrictive physiology, integrating echocardiography, CT, and CMR with microbiological confirmation. This case illustrates how multimodality imaging may help characterize an active inflammatory constrictive phenotype and guide therapeutic decision-making, while the potential reversibility of constrictive physiology requires assessment during follow-up.

## Case presentation

A 20-year-old male university student with no significant medical history presented to a cardiology consultation with a one-month history of progressive unintentional weight loss, profuse night sweats, and intermittent predominantly nocturnal fever. He had received Bacillus Calmette-Guérin (BCG) vaccination and reported no known contact with a patient with active tuberculosis. There was no history of recent travel, immunosuppression, chronic systemic disease, or other identifiable risk factor for tuberculosis.

His symptoms were associated with chest pain exhibiting typical pericarditic features. The pain was sharp and pleuritic, worsened by deep inspiration and the supine position, and partially relieved by sitting upright and leaning forward. He denied syncope, presyncope, palpitations, or exertional angina. There were no respiratory symptoms suggestive of active pulmonary involvement, including chronic cough, hemoptysis, or significant sputum production. The infectious history was otherwise unremarkable, with no recent viral syndrome or documented bacterial infection.

On admission, the patient was febrile, with a temperature of 38.5°C. He was hemodynamically stable, with a blood pressure of 120/80 mmHg and a regular heart rate of 80 beats/min. Cardiac auscultation revealed muffled heart sounds, with no pericardial friction rub, pericardial knock, gallop rhythm, or cardiac murmur. Clinical examination showed signs of systemic venous congestion, with bilateral pitting lower-limb edema predominantly involving the ankles and distal legs. Pulmonary auscultation revealed clear breath sounds bilaterally, without crackles. There was no clinically detectable hepatomegaly or splenomegaly and no palpable peripheral lymphadenopathy. Both calves and thighs were soft and non-tender, without clinical signs suggestive of deep-vein thrombosis.

A 12-lead electrocardiogram showed a regular sinus rhythm at 80 beats/min, with a constant PR interval and narrow QRS complexes. No significant repolarization abnormalities were observed, and there were no ST-segment or T-wave changes suggestive of acute pericarditis. Overall, the electrocardiogram was unremarkable.

Chest radiography revealed enlargement of the cardiopericardial silhouette. No visible pericardial calcifications or focal pulmonary parenchymal abnormalities were identified (Figure 1).

**Figure 1:**
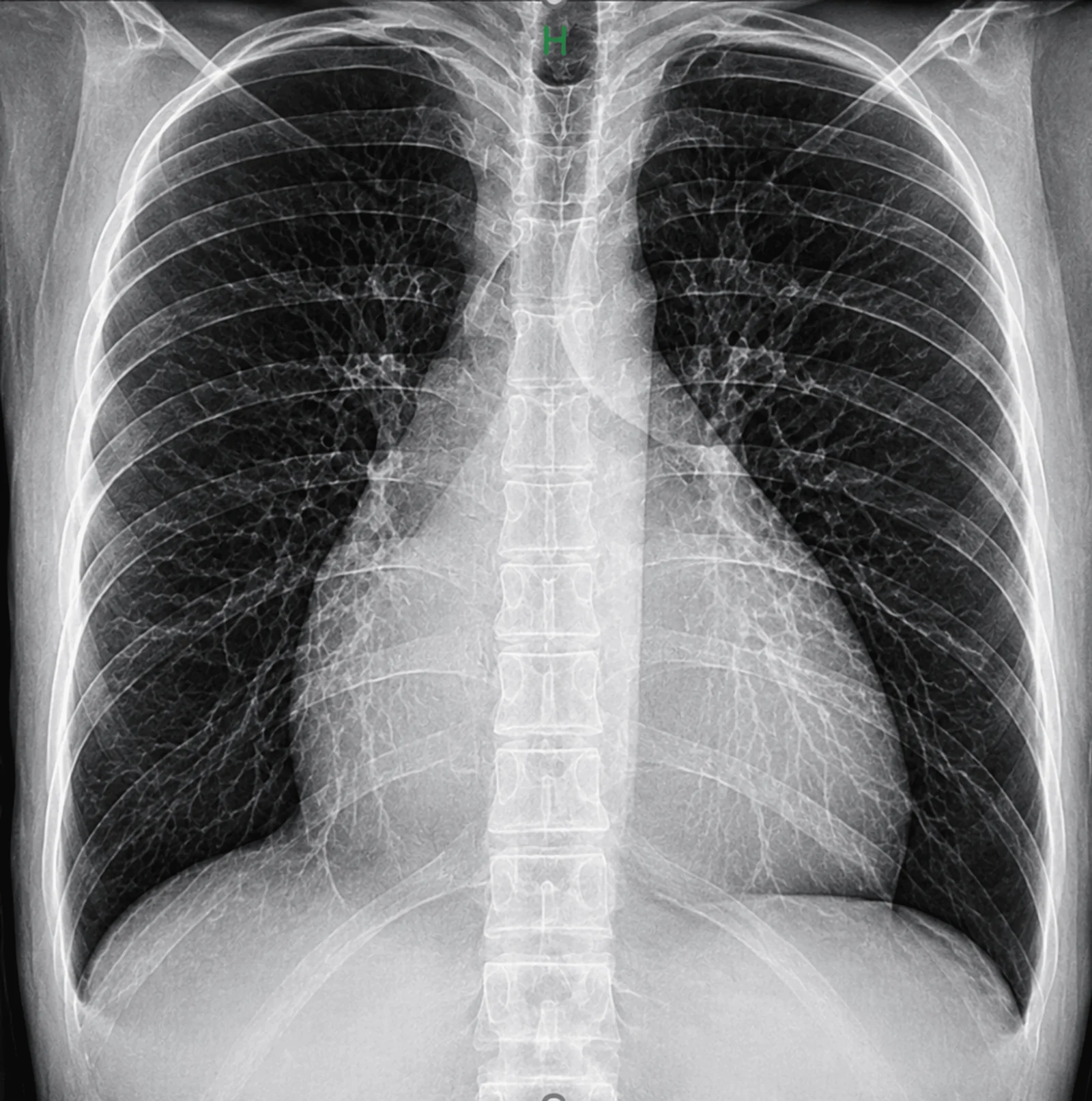
Chest radiograph showing cardiopericardial silhouette enlargement Chest radiograph showing an enlarged cardiopericardial silhouette, without visible pericardial calcification or focal pulmonary parenchymal abnormalities.

Transthoracic echocardiography (TTE) revealed marked thickening of both pericardial layers, measuring 7 mm for the parietal pericardium and 6 mm for the visceral pericardium (Figure 2).

**Figure 2:**
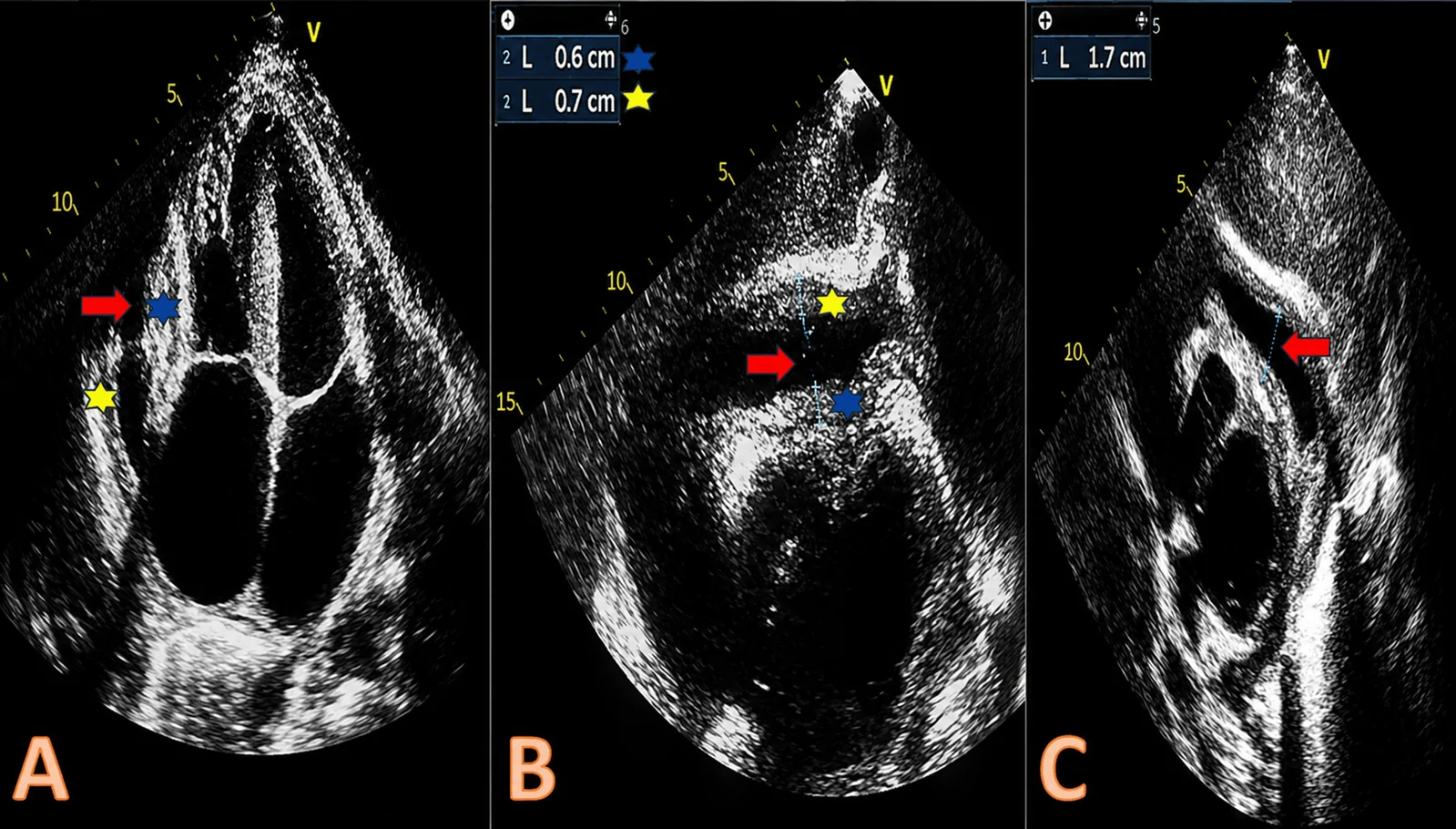
Transthoracic echocardiographic findings of pericardial involvement. (A) Apical four-chamber view showing thickening of the parietal pericardium (yellow star) and visceral pericardium (blue star), with associated pericardial effusion (red arrow); (B) Subcostal view demonstrating marked thickening of the parietal pericardium (yellow star, 7 mm) and visceral pericardium (blue star, 6 mm), with associated pericardial effusion (red arrow); (C) Subcostal view showing pericardial effusion adjacent to the cardiac apex, with a maximal end-diastolic separation of 17 mm (red arrow).

A moderate circumferential pericardial effusion was present, with a maximal end-diastolic separation of 17 mm along the right ventricular free wall and at the apex, and 17 mm along the left ventricular lateral wall.

Several echocardiographic features of constrictive physiology were identified. Abnormal interventricular septal motion with a characteristic paradoxical septal shift (“septal bounce”) was observed. Pulsed-wave Doppler assessment of mitral inflow showed an E-wave velocity of 0.58 m/s and an A-wave velocity of 0.36 m/s, yielding an E/A ratio of 1.77. Respiratory variation in transmitral E-wave velocity was approximately 25% (Figure 3A), reaching the conventional threshold suggestive of constrictive physiology and, in conjunction with the other echocardiographic findings, supporting enhanced ventricular interdependence.

**Figure 3:**
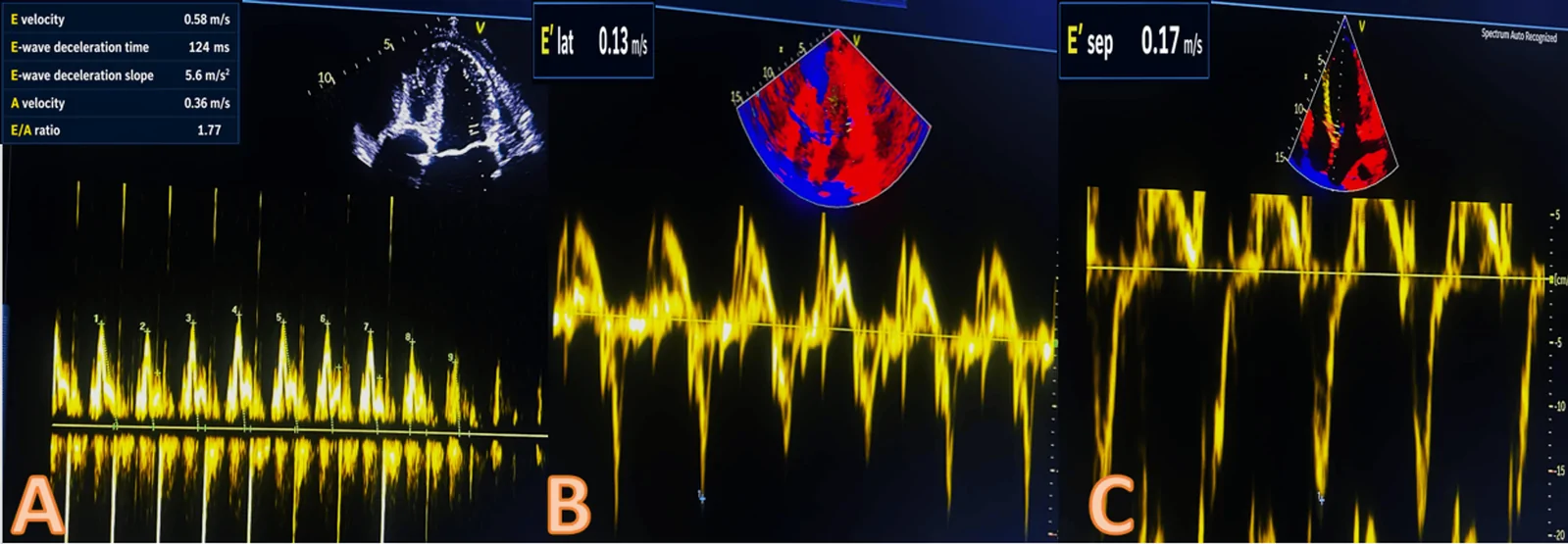
Doppler echocardiographic features of constrictive physiology (A) Pulsed-wave Doppler of transmitral inflow showing an E-wave velocity of 0.58 m/s, an A-wave velocity of 0.36 m/s, and an E/A ratio of 1.77, with approximately 25% respiratory variation in E-wave velocity, a borderline finding considered supportive of constrictive physiology; (B–C) Tissue Doppler imaging of the mitral annulus showing a lateral e′ velocity of 0.13 m/s (B) and a septal e′ velocity of 0.17 m/s (C). The higher septal than lateral e′ velocity demonstrates annulus reversus, while the preserved septal e′ velocity with a low E/e′ ratio is consistent with annulus paradoxus. Together with the other echocardiographic findings, these features support constrictive physiology.

Tissue Doppler imaging demonstrated preserved mitral annular early diastolic velocities, with a septal e′ velocity of 0.17 m/s and a lateral e′ velocity of 0.13 m/s. The higher septal than lateral e′ velocity was consistent with the characteristic annulus reversus pattern (Figures 3B, 3C). Despite the constrictive physiology, the septal E/e′ ratio remained low at approximately 3.4, illustrating the annulus paradoxus phenomenon.

The left ventricle was non-dilated, with normal wall thickness and preserved global systolic function (left ventricular ejection fraction or LVEF, 60%). The right ventricle was also non-dilated, with preserved systolic function, and both atria were normal in size. The inferior vena cava was dilated to 25 mm with reduced respiratory collapsibility, consistent with elevated right atrial pressure. Trivial tricuspid regurgitation was present, with a peak tricuspid regurgitation velocity of 2.0 m/s.

Overall, although the approximately 25% respiratory variation in transmitral E-wave velocity was borderline and therefore considered a supportive rather than isolated diagnostic finding, the combination of marked pericardial thickening, circumferential pericardial effusion, septal bounce, preserved septal e′ velocity, annulus reversus, annulus paradoxus, and a dilated, poorly collapsible inferior vena cava strongly supported the presence of pericardial effusion with echocardiographic evidence of constrictive physiology.

Given the clinical presentation and echocardiographic evidence of pericardial effusion with constrictive physiology, the patient was admitted to the cardiology department for further etiological investigation and management. Contrast-enhanced chest CT was subsequently performed to further characterize the pericardial involvement and assess for associated thoracic abnormalities. CT demonstrated diffuse circumferential pericardial thickening associated with a moderate circumferential pericardial effusion (Figure 4).

**Figure 4:**
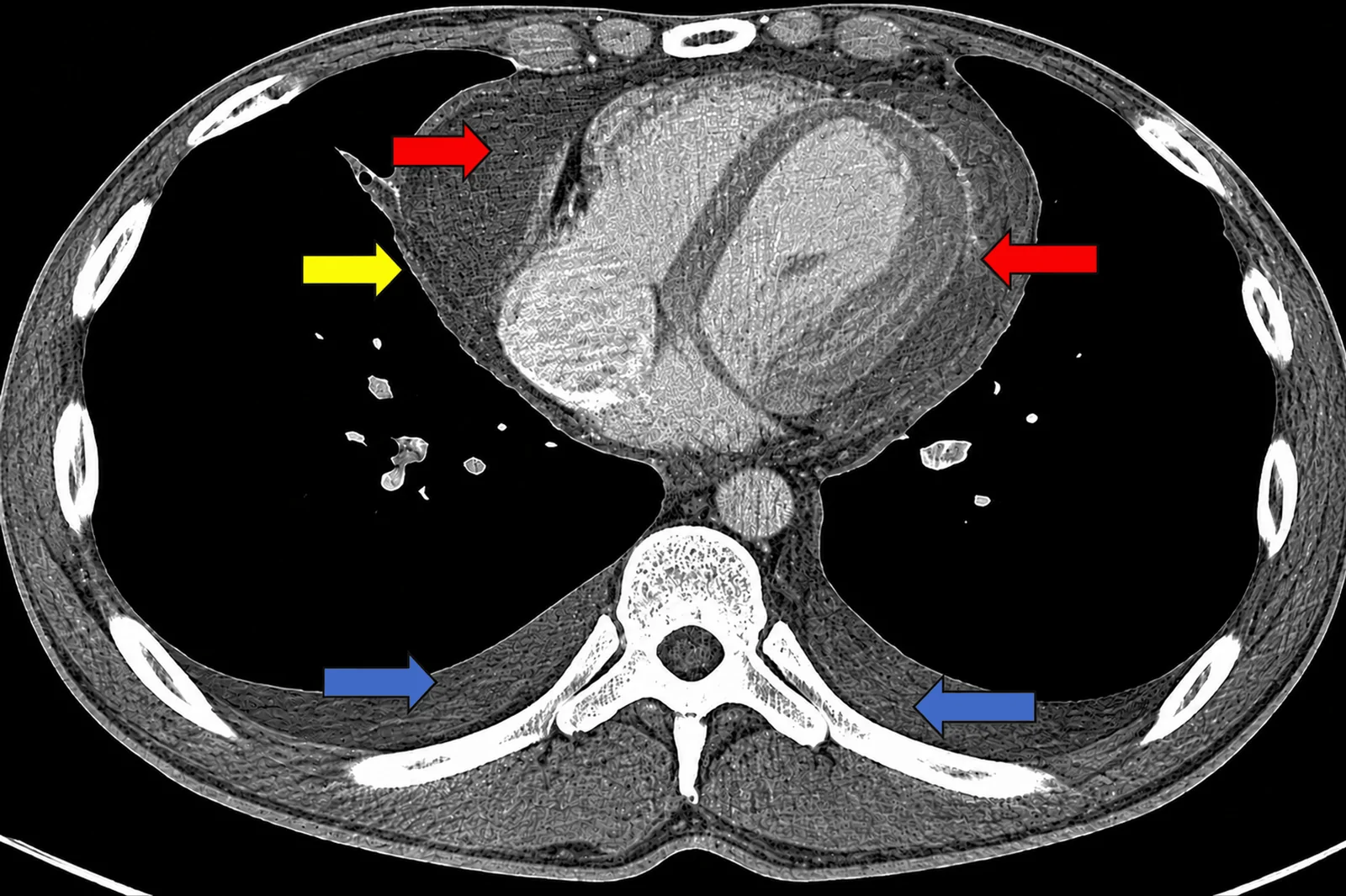
Contrast-enhanced CT demonstrating pericardial thickening and effusion. Contrast-enhanced axial chest computed tomography (CT) showing a circumferential pericardial effusion (red arrows) associated with pericardial thickening (yellow arrow). Small bilateral pleural effusions are also present (blue arrows). No visible pericardial calcification is identified.

Small bilateral pleural effusions were also present. No pericardial calcifications or focal pulmonary parenchymal abnormalities were identified, and there was no evidence of pulmonary embolism.

These findings confirmed the morphological pericardial abnormalities identified on echocardiography and, in the clinical context, further supported an active inflammatory pericardial process.

CMR imaging was subsequently performed for further morphological and tissue characterization of the pericardium. Cine sequences confirmed a moderate circumferential pericardial effusion associated with marked diffuse thickening of both the visceral and parietal pericardial layers (Figure 5).

**Figure 5:**
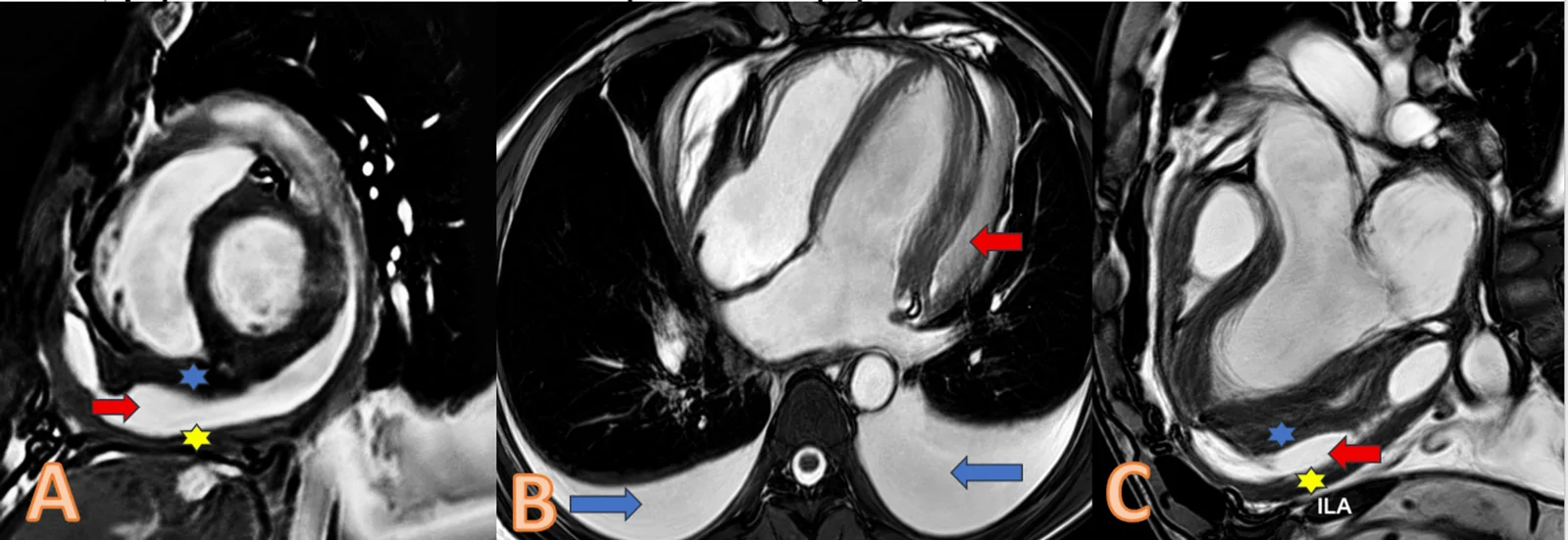
Cardiac magnetic resonance imaging findings of pericardial involvement Cine cardiac magnetic resonance (CMR) sequences in the (A) short-axis, (B) four-chamber, and (C) three-chamber views demonstrating a circumferential pericardial effusion (red arrows) associated with marked thickening of the visceral (blue stars) and parietal (yellow stars) pericardial layers. Bilateral pleural effusions are also visible in the four-chamber view (B, blue arrows).

Bilateral pleural effusions were also noted. Cardiac chamber dimensions were preserved, without significant ventricular dilatation.

Late gadolinium enhancement (LGE) imaging demonstrated intense diffuse enhancement of the thickened pericardium, consistent with active pericardial inflammation, while the intervening pericardial effusion remained hypointense (Figure 6).

**Figure 6:**
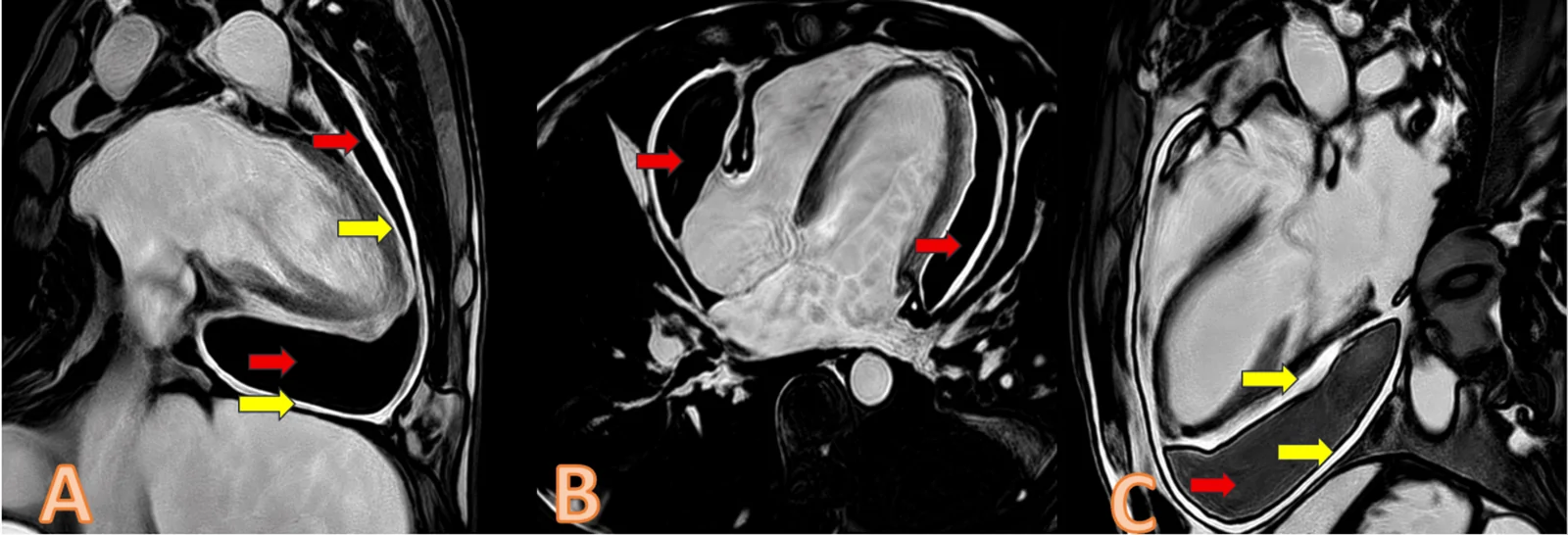
Late gadolinium enhancement cardiac magnetic resonance findings of active pericardial inflammation Late gadolinium enhancement (LGE) images in the (A) two-chamber, (B) four-chamber, and (C) three-chamber views demonstrate intense enhancement of the thickened pericardium (yellow arrows), consistent with active pericardial inflammation, associated with a circumferential hypointense pericardial effusion (red arrows).

The combination of diffuse pericardial thickening, circumferential effusion, and marked pericardial LGE supported an active inflammatory pericardial process. Together with the echocardiographic evidence of constrictive physiology, these CMR findings supported the presence of pericardial effusion with constrictive physiology associated with active pericardial inflammation.

Laboratory investigations revealed a marked inflammatory response, with mild leukocytosis (11,000/µL) with lymphocyte predominance, a C-reactive protein (CRP) level of 100 mg/L, an erythrocyte sedimentation rate (ESR) of 75 mm/h, and a fibrinogen level of 5.8 g/L. Serum protein electrophoresis showed an inflammatory pattern. Hemoglobin and platelet counts were within normal limits. Serum creatinine was within the normal range (8 mg/L), with an estimated glomerular filtration rate (eGFR) of 80 mL/min/1.73 m². Hepatic function, electrolytes, albumin, lactate dehydrogenase (LDH), and high-sensitivity cardiac troponin were within normal ranges, whereas N-terminal pro-B-type natriuretic peptide (NT-proBNP) was elevated at 1,000 pg/mL. Thyroid function and the coagulation profile were unremarkable (Table 1).

**Table 1:** Hematological, inflammatory, metabolic, and cardiac laboratory findings aPTT, activated partial thromboplastin time; AST, aspartate aminotransferase; ALT, alanine aminotransferase; CRP, C-reactive protein; eGFR, estimated glomerular filtration rate; ESR, erythrocyte sedimentation rate; INR, international normalized ratio; LDH, lactate dehydrogenase; NT-proBNP, N-terminal pro-B-type natriuretic peptide; PT, prothrombin time; TSH, thyroid-stimulating hormone.

Laboratory parameter	Patient result	Reference range
Hematology and inflammatory profile		
Hemoglobin	14 g/dL	13–17 g/dL
White blood cell count	11,000/µL	4,000–10,000/µL
Neutrophils	38% (4,180/µL)	40–70%
Lymphocytes	52% (5,720/µL)	20–40%
Monocytes	7% (770/µL)	2–10%
Eosinophils	2% (220/µL)	1–6%
Basophils	1% (110/µL)	0–1%
Platelet count	250,000/µL	150,000–400,000/µL
C-reactive protein (CRP)	100 mg/L	<5 mg/L
Erythrocyte sedimentation rate (ESR)	75 mm/h	<15 mm/h
Fibrinogen	5.8 g/L	2.0–4.0 g/L
Serum protein electrophoresis	Inflammatory pattern	Normal pattern
Renal and metabolic profile		
Urea	0.30 g/L	0.15–0.45 g/L
Serum creatinine	8 mg/L	6–12 mg/L
eGFR	80 mL/min/1.73 m²	≥90 mL/min/1.73 m²
Sodium	138 mmol/L	135–145 mmol/L
Potassium	4.2 mmol/L	3.5–5.0 mmol/L
Liver and cardiac biomarkers		
AST	35 U/L	<40 U/L
ALT	35 U/L	<40 U/L
Albumin	45 g/L	35–50 g/L
LDH	200 U/L	135–225 U/L
High-sensitivity troponin	8 ng/L	<14 ng/L
NT-proBNP	1,000 pg/mL	<125 pg/mL
Thyroid function		
TSH	1.8 mIU/L	0.4–4.0 mIU/L
Hemostasis		
Prothrombin time (PT)	12.5 s	11–14 s
Prothrombin activity	95%	70–100%
INR	1	0.8–1.2
aPTT	30 s	25–35 s

An extensive etiological work-up was performed. Interferon-gamma release assay (IGRA) was positive, whereas testing for human immunodeficiency virus (HIV), hepatitis B virus (HBV), and hepatitis C virus (HCV) was negative (Table 2).

**Table 2:** Tuberculosis and infectious work-up AFB, acid-fast bacilli; HBsAg, hepatitis B surface antigen; HCV, hepatitis C virus; HIV, human immunodeficiency virus; IGRA, interferon-gamma release assay; MTB, Mycobacterium tuberculosis; Xpert MTB/RIF, molecular assay for detection of Mycobacterium tuberculosis and rifampicin resistance.

Laboratory parameter	Patient result	Reference/expected result
IGRA (QuantiFERON-TB)	Positive	Negative
Tuberculin skin test	Positive	Negative
Pericardial fluid Xpert MTB/RIF	MTB detected	MTB not detected
Rifampicin resistance	Not detected	Not detected
Pericardial fluid mycobacterial culture	Positive for M. tuberculosis	Negative
Pericardial fluid AFB smear	Negative	Negative
Sputum AFB smear	Negative	Negative
Sputum Xpert MTB/RIF	MTB not detected	MTB not detected
Blood cultures	Negative	Negative
HIV serology	Negative	Negative
HBsAg	Negative	Negative
Anti-HCV antibodies	Negative	Negative

Autoimmune screening, including antinuclear antibodies (ANA), anti-double-stranded DNA (anti-dsDNA) antibodies, antineutrophil cytoplasmic antibodies (ANCA), rheumatoid factor (RF), anti-cyclic citrullinated peptide (anti-CCP) antibodies, and complement C3 and C4 levels, was unremarkable. Screening for alternative etiologies included carcinoembryonic antigen (CEA), alpha-fetoprotein (AFP), beta-human chorionic gonadotropin (β-hCG), and carbohydrate antigen (CA 19-9), all of which were within normal ranges (Table 3).

**Table 3:** Autoimmune and malignancy work-up AFP, alpha-fetoprotein; ANA, antinuclear antibodies; ANCA, antineutrophil cytoplasmic antibodies; anti-CCP, anti-cyclic citrullinated peptide antibodies; anti-dsDNA, anti-double-stranded DNA antibodies; β-hCG, beta-human chorionic gonadotropin; CA 19-9, carbohydrate antigen 19-9; CEA, carcinoembryonic antigen.

Laboratory parameter	Patient result	Reference/expected result
Autoimmune work-up		
Antinuclear antibodies (ANA)	Negative	Negative
Anti-dsDNA antibodies	Negative	Negative
ANCA	Negative	Negative
Rheumatoid factor	Negative	Negative
Anti-CCP antibodies	Negative	Negative
Complement C3	1.20 g/L	0.90–1.80 g/L
Complement C4	0.25 g/L	0.10–0.40 g/L
Malignancy work-up		
CEA	1.8 ng/mL	<5 ng/mL
AFP	3.2 ng/mL	<10 ng/mL
β-hCG	<2 IU/L	<5 IU/L
CA 19-9	12 U/mL	<37 U/mL
Pericardial fluid cytology	Negative for malignant cells	Negative

Pericardiocentesis was performed for etiological assessment, yielding 200 mL of citrine-yellow pericardial fluid. Biochemical analysis demonstrated an exudative profile, with a pericardial fluid-to-serum protein ratio of 0.7. Cellular analysis showed lymphocytic predominance, and the pericardial fluid adenosine deaminase (ADA) level was markedly elevated at 100 U/L. Xpert MTB/RIF (Cepheid, Sunnyvale, CA, USA) testing of the pericardial fluid detected Mycobacterium tuberculosis without rifampicin resistance, and subsequent mycobacterial culture was positive for M. tuberculosis, establishing a definitive microbiological diagnosis of tuberculous pericarditis. Routine bacterial cultures were negative (Table 2), and pericardial fluid cytology showed no malignant cells (Table 3).

The case was discussed by a multidisciplinary team involving cardiologists, pulmonologists, infectious disease specialists, and cardiothoracic surgeons. Given the microbiologically confirmed TBP associated with pericardial effusion and constrictive physiology, the decision was made to initiate a six-month course of standard antituberculous therapy, consisting of rifampicin, isoniazid, pyrazinamide, and ethambutol during the initial two-month intensive phase, followed by rifampicin and isoniazid for an additional four months. Given the marked inflammatory pericardial involvement and the patient’s HIV-negative status, adjunctive oral prednisolone was initiated at 120 mg/day for one week, followed by a gradual six-week taper consisting of 90 mg/day, 60 mg/day, 30 mg/day, 15 mg/day, and 5 mg/day, each administered for one week before discontinuation.

Considering the prominent active inflammatory component demonstrated by CMR imaging, an initial medical strategy was adopted, with close clinical, laboratory, echocardiographic, and imaging follow-up. Pericardiectomy was reserved for persistent or progressive constrictive physiology despite adequate antituberculous and anti-inflammatory therapy, with its indication to be reassessed according to the patient’s clinical and imaging evolution.

The diagnosis, therapeutic strategy, potential complications, and prognosis were thoroughly explained to the patient. At short-term follow-up, the patient experienced symptomatic improvement, accompanied by a decrease in CRP from 100 mg/L to 50 mg/L. Repeat TTE demonstrated a reduction in pericardial effusion; however, echocardiographic features of constrictive physiology persisted. Longer-term clinical and imaging follow-up was not yet available at the time of manuscript preparation.

## Discussion

TBP is a clinically significant and potentially severe manifestation of M. tuberculosis infection, accounting for approximately 1% of all tuberculosis cases and 1-2% of extrapulmonary tuberculosis [4]. Its contribution to pericardial disease varies considerably according to geographical setting and tuberculosis prevalence. In endemic regions, TBP may account for 50-90% of effusive pericarditis, compared with approximately 4% in non-endemic regions [4]. Although more frequently encountered in endemic settings and immunocompromised populations, TBP can also affect young immunocompetent patients without known tuberculosis exposure or overt pulmonary disease, as illustrated by our case [2,4].

TBP represents a dynamic inflammatory process encompassing a spectrum from acute pericarditis and pericardial effusion to constrictive physiology and, ultimately, chronic constrictive pericarditis. Pericardial effusion is the most frequent presentation, occurring in up to 80% of cases [1]. In our patient, the coexistence of a moderate circumferential effusion with multiple echocardiographic features of constriction supported the presence of pericardial effusion with echocardiographic evidence of constrictive physiology. The constitutional syndrome characterized by fever, weight loss, and profuse night sweats, despite the absence of overt pulmonary involvement, raised suspicion of an underlying chronic infectious process.

Multimodality imaging was central to characterizing both the anatomical and functional components of pericardial involvement. Echocardiography demonstrated pericardial thickening and effusion together with septal bounce, approximately 25% respiratory variation in transmitral E-wave velocity, preserved septal e′ velocity, annulus reversus, and annulus paradoxus. Although the respiratory variation in transmitral flow was borderline and therefore considered supportive rather than diagnostic in isolation, the combination of these findings supported enhanced ventricular interdependence and constrictive physiology [5,6]. The diagnostic relevance of these echocardiographic parameters is supported by a meta-analysis of 17 studies including 889 patients, in which ventricular septal shift, respiratory variation in mitral inflow, preserved septal e′ velocity, and an increased septal-to-lateral e′ ratio demonstrated good diagnostic performance for constrictive pericarditis [5].

An important differential diagnosis is restrictive cardiomyopathy (RCM), since both conditions may present with preserved left ventricular systolic function, impaired diastolic filling, elevated filling pressures, and systemic venous congestion. Their underlying mechanisms, however, differ fundamentally: constrictive pericarditis results from external limitation of cardiac filling by a noncompliant pericardium, whereas RCM results from intrinsic myocardial dysfunction [5,6]. In RCM, mitral annular e′ velocities are generally reduced because myocardial relaxation is impaired. Conversely, septal e′ is typically preserved or increased in constrictive pericarditis, and a septal e′ velocity exceeding the lateral e′ produces annulus reversus. Respiratory ventricular septal shift and respiratory variation in transmitral flow may further support constrictive physiology [5,6]. In our patient, a preserved septal e′ velocity of 17 cm/s exceeding the lateral e′ velocity of 13 cm/s, together with septal bounce and annulus reversus, strongly favored constrictive rather than restrictive physiology. The approximately 25% respiratory variation in transmitral flow provided additional supportive evidence but was considered borderline in isolation. In diagnostically equivocal cases, simultaneous right- and left-heart catheterization may provide additional discrimination [1].

Other differential diagnoses of pericardial effusion and constriction include idiopathic or viral pericarditis, bacterial infection, autoimmune or systemic inflammatory diseases, malignancy, uremia, hypothyroidism, previous cardiac surgery, and radiation-induced pericardial disease [1]. In the present case, serum creatinine within the normal range, normal thyroid function, negative autoimmune testing, negative HIV and viral hepatitis serologies, negative routine bacterial cultures, and the absence of malignant cells in the pericardial fluid made these alternative etiologies less likely. Most importantly, Xpert MTB/RIF detected M. tuberculosis in the pericardial fluid without rifampicin resistance, and subsequent mycobacterial culture was positive, establishing a definitive microbiological diagnosis of tuberculous pericarditis. These findings are particularly important because neither clinical presentation nor multimodality imaging alone can establish the tuberculous etiology [1].

CT and CMR provided complementary anatomical and tissue characterization. CT confirmed diffuse pericardial thickening and circumferential effusion without calcification, emphasizing that the absence of pericardial calcification does not exclude constrictive physiology [3]. CMR was particularly informative, demonstrating intense LGE of the thickened pericardium, consistent with ongoing active inflammation [1,3]. This distinction has important therapeutic implications, as constrictive physiology associated with active inflammation may indicate a potentially reversible inflammatory component rather than established irreversible fibrosis [1,3]. However, the presence of active inflammation alone does not establish reversibility, which requires assessment during follow-up. The 2025 European Society of Cardiology (ESC) guidelines recognize transient constriction as a potentially reversible condition and emphasize the value of inflammatory biomarkers and CMR in identifying ongoing pericardial inflammation [1].

Treatment of TBP is primarily based on appropriate antituberculous chemotherapy. Current ESC recommendations support a six-month multidrug regimen, generally consisting of rifampicin, isoniazid, pyrazinamide, and ethambutol for the first two months, followed by rifampicin and isoniazid for an additional four months [1]. Adjunctive corticosteroid therapy may be considered in HIV-negative patients [1]. In the randomized Investigation of the Management of Pericarditis (IMPI) trial, prednisolone did not significantly reduce the composite primary outcome of death, cardiac tamponade, or constrictive pericarditis, although it reduced the incidence of constrictive pericarditis from 7.8% to 4.4% and was associated with fewer hospitalizations [7]. In light of these findings, adjunctive corticosteroid therapy was considered a reasonable therapeutic option in our HIV-negative patient with marked active pericardial inflammation, while acknowledging that the available evidence does not establish a definitive overall outcome benefit [1,7].

The prognosis of TBP is closely related to immune status, the severity of pericardial involvement, and progression toward irreversible constriction. TBP remains associated with substantial morbidity and mortality, with reported six-month mortality ranging from approximately 17% to 40% [1]. Before effective antituberculous therapy, constriction developed in up to 50% of patients within six months, whereas contemporary treatment has substantially reduced this risk [1]. Persistent or progressive constrictive physiology despite adequate antituberculous and anti-inflammatory therapy may suggest evolution toward fixed fibrotic constriction and warrant consideration of pericardiectomy [1,8].

In our patient, the combination of young age, HIV-negative status, preserved biventricular systolic function, absence of pericardial calcification, and, particularly, marked inflammatory activity on CMR suggests an active inflammatory phenotype in which a potentially reversible component of constriction may still be present [1,3]. These features should not, however, be interpreted as demonstrating reversibility. At short-term follow-up, the patient experienced symptomatic improvement, accompanied by a decrease in CRP from 100 mg/L to 50 mg/L and a reduction in pericardial effusion. Nevertheless, echocardiographic features of constrictive physiology persisted. These early findings suggest a partial clinical and inflammatory response to therapy but do not establish reversibility of the constrictive physiology. Continued clinical and multimodality imaging surveillance is therefore warranted to determine whether constrictive physiology subsequently resolves with medical therapy or persists, potentially requiring pericardiectomy [1,3,8]. Longer-term clinical and imaging outcomes were not yet available at the time of manuscript preparation.

## Conclusions

TBP remains a challenging and potentially severe manifestation of extrapulmonary tuberculosis. The particular interest of this case lies in the occurrence of microbiologically confirmed TBP in a young immunocompetent patient with no known tuberculosis exposure or overt pulmonary involvement, presenting with pericardial effusion and echocardiographic evidence of constrictive physiology. Multimodality imaging provided a comprehensive and complementary assessment: echocardiography identified the characteristic physiological features of constriction, CT delineated the extent of pericardial involvement, and CMR demonstrated marked active pericardial inflammation.

Definitive etiological diagnosis was established by detection of M. tuberculosis using Xpert MTB/RIF and subsequent positive mycobacterial culture of the pericardial fluid. Beyond diagnosis, this case emphasizes the importance of recognizing an active inflammatory constrictive phenotype, which may include a potentially reversible component and therefore inform therapeutic decision-making. However, active inflammation does not establish reversibility or demonstrate that treatment will prevent progression to fixed fibrotic constriction. Early recognition, microbiological confirmation, and close clinical and multimodality imaging follow-up are therefore essential to guide management and determine the subsequent evolution of constrictive physiology.
